# Medicinally important aromatic plants with radioprotective activity

**DOI:** 10.4155/fsoa-2017-0061

**Published:** 2017-09-21

**Authors:** Ravindra M Samarth, Meenakshi Samarth, Yoshihisa Matsumoto

**Affiliations:** 1 Department of Research, Bhopal Memorial Hospital & Research Centre, Department of Health Research, Government of India, Raisen Bypass Road, Bhopal 462038, India; 2 ICMR-National Institute for Research in Environmental Health, Kamla Nehru Hospital Building, GMC Campus, Bhopal 462001, India; 3 Faculty of Science, RKDF University, Airport Bypass Road, Gandhi Nagar, Bhopal 462033, India; 4 Tokyo Institute of Technology, Institute of Innovative Research, Laboratory for Advanced Nuclear Energy, N1–30 2–12–1 Ookayama, Meguro-ku, Tokyo 152–8550, Japan

**Keywords:** antioxidant activity, aromatic plants, essential oils, lymphocytes, mice, radiation protection, radical scavenging activity, rat

## Abstract

Aromatic plants are often used as natural medicines because of their remedial and inherent pharmacological properties. Looking into natural resources, particularly products of plant origin, has become an exciting area of research in drug discovery and development. Aromatic plants are mainly exploited for essential oil extraction for applications in industries, for example, in cosmetics, flavoring and fragrance, spices, pesticides, repellents and herbal beverages. Although several medicinal plants have been studied to treat various conventional ailments only a handful studies are available on aromatic plants, especially for radioprotection. Many plant extracts have been reported to contain antioxidants that scavenge free radicals produced due to radiation exposure, thus imparting radioprotective efficacy. The present review focuses on a subset of medicinally important aromatic plants with radioprotective activity.

## Lay abstract

Aromatic plants have been used as natural medicines since prehistoric times. They are currently mainly utilized for essential oil extraction and are widely used in cosmetics, flavoring and fragrance, spices, pesticides, repellent and herbal beverages. Several medicinal plants have shown promise for the treatment various diseases including cancer. However, only a handful studies are available on aromatic plants, especially in terms of radioprotection. The present review focuses on certain medicinally important aromatic plants with special reference to their radioprotective effects.

Plants with medicinal or aromatic properties that are used in pharmacy and/or perfumery are usually defined as medicinal and aromatic plants; however, medicinal, aromatic and cosmetic plants would be a better term as many medicinal and aromatic plants are also used in cosmetics [1]. Aromatic plants are those that contain aromatic compounds – basically essential oils that are volatile at room temperature. These essential oils are odorous, volatile, hydrophobic and highly concentrated compounds. They can be obtained from flowers, buds, seeds, leaves, twigs, bark, wood, fruits and roots [2]. Essential oils are complex mixtures of secondary metabolites comprised low-boiling-point phenylpropenes and terpenes [3]. These oils usually consist of a about tens-to-hundreds of low molecular weight terpenoids. Even unidentified trace constituents may be held responsible for altering the odour, flavour and the bioactivity of the oil to a considerable degree. Essential oils have characteristic flavor and fragrance properties, possess biological activities and are widely applied in aromatherapy and healthcare in addition to several industries such as cosmetics, flavoring and fragrance, spices, pesticides and repellents, as well as herbal beverages.

Antioxidant and antimicrobial activities of aromatic plants have been widely explored and found to have health applications in prevention and reducing risk of diseases such as inflammation, atherosclerosis, cardiovascular and cancer [4,5]. Various plant families, particularly *Lamiaceae* [6–19], *Apiaceae* [20–23] and *Zinziberaceae* [24–28] have been investigated in depth for their medicinal value due to their significant antioxidant properties. The antioxidant activities of aromatic plants is influenced by various factors such as growing conditions, methods of processing/extraction and importantly constituents of the antioxidants [29]; the methods involved in determination of antioxidant capacity as well as extraction therefore play a crucial role [30].

It is a well-known fact that radiation is a powerful cytotoxic agent. Reactive oxygen species such as superoxide anion, singlet oxygen, hydroxyl radical, nitric oxide, hydrogen peroxide and peroxyl radicals are generated by ionizing radiation in biologic system through radiolysis of water and are accountable for cellular injury caused to DNA and proteins [31–33]. Free radicals generated through ionizing radiation attack DNA, producing single strand breaks and double strand breaks (DSBs) due to oxidative insult to sugar moiety and base components [33,34]. Unrepaired or misrepaired DSBs eventually lead to the chromosomal aberrations, ultimately paving the way to mutagenesis, carcinogenesis and hereditary diseases. Thus, DSBs are considered as the most vulnerable radiation-induced DNA damage. Homologous recombination and nonhomologous end joining are major pathways involved in repair of DSB in eukaryotic cells [35]. In the nonhomologous end joining pathway, DNA-dependent protein kinase (DNA-PK), which consists of DNA-PKcs (DNA-PK catalytic subunit) and Ku70/Ku80 (also known as Ku86) heterodimer, acts as the molecular sensor for DSBs. DSBs are finally sealed by DNA ligase IV, in association with XRCC4 and XLF (also known as Cernunnos). DNA-PK phosphorylates a number of proteins, including XRCC4 in response to radiation [36,37]. Although the role of phosphorylation of XRCC4 in DSB repair remains to be clarified, the phosphorylation status of XRCC4 will serve as marker of DNA-PK functionality in living cells.

Thus, the detrimental effects of radiation-induced alterations in biologic systems via reactive oxygen species generation play a crucial role in maintenance of metabolic homeostasis in the body. Therefore, any disparity in homeostasis results in oxidative stress [38], which can be trounced by additional provision of naturally occurring, plant based antioxidants [39–41]. It has been shown that the oxidation process could be halted by antioxidants by adopting various strategies such as scavenging, chelating or transferring hydrogen atoms [42–44]. It has been speculated that the radioprotectors must have radical-scavenging properties along with antioxidant function; however, all antioxidants do not provide radioprotection [45]. Radioprotectors minimize or reduce the radiation-induced damage to normal tissues and need to be present before or at the time of radiation exposure for its protective efficacy [46]. In recent years, investigations have demonstrated the importance and usefulness of aromatic plants for their radioprotective effects and ability to be employed for modification of oxidative insult due to radiation. Therefore, the present review focused on medicinally important aromatic plants with special reference to radioprotective activity (Table 1).

## Radioprotection by medicinal aromatic plants

### *Ageratum conyzoides*

The plant *Ageratum* belongs to the family Asteraceae, which consists of about 30 species. The name is derived from the Greek word ‘a geras’ and konyz, referring to long life and similarity with *Inula helenium*. It is an aromatic herb, erect, annual and growing up to 1 m in height, commonly found in Africa, Asia, America and Australia. Few species of *Ageratum* have been pharmacologically evaluated. The weed is best known for its healing activity and has been used for treating burn, wound, skin diseases and various infections among others, since ancient times [47]. *Ageratum conyzoides* contains various chemical constituents [48–55]. It is rich in polyoxygenated flavonoids. The triterpenes are friedelin, and the major sterols are sterols-β-sitosterol and stigmasterol. Lycopsamine and echinatine are two isomeric pyrrolizidine alkaloids isolated from *A. conyzoides*. The oil content varies from 0.11–0.58% for leaves and from 0.03–0.18% for the roots. The major components of the essential oil are 7-methoxy-2,2-dimethylchromene (prescocene I), 6,7-dimethoxy derivative, ageratochromene (precocene II) and ageratochromene dimmer.

**Table 1.** **Summary of aromatic plants investigated for their radioprotective activity and their chemical constituents.**

**Name of aromatic plant**	**Plant part used**	**Doses**		**Test system and parameters studied**	**Suggested possible mechanism**	**Chemical constituents**	**Ref.**

		**Plant extract**	**Radiation**				
*Ageratum conyzoides*	Whole plant extract	75 mg/kg	10 Gy	Mice/survival assay	Free radical scavenging	It is rich in polyoxygenated flavonoids. The triterpenes are friedelin, and the major sterols are sterols-β-sitosterol and stigmasterol Lycopsamine and echinatine, two isomeric pyrrolizidine are the alkaloids isolated from *A. conyzoides.* The oil content varies randomly from 0.11–0.58% for leaves and from 0.03–0.18% for the roots. The major components of the essential oil are 7-methoxy-2,2-dimethylchromene (precocene I), 6,7-dimethoxy derivative, ageratochromene (precocene II) and ageratochromene dimmer	[48–56]

*Allium cepa*	Bulb	5 ml/kg	X-ray 525 kv/s	Rats/biochemical parameters	Anti-oxidative and free radical scavenging	The essential oil of *Allium cepa* contains compounds such as 3-1,8-cineole, L-linalool and camphordare The onion bulb contains Kaempferol, β-sitosterol, ferulic acid, myritic acid and prostaglandins. Flavonoids and tannins also present in *A. cepa.* Quercetin, quercetin 4-glucoside, taxifolin, taxifolin 7-glucoside and phenylalanine have been isolated from the bulb The major sulfur compounds are dimethyl trisulfide, propenyl propyl disulfide, dipropyl disulfide, propenylmethyldisulfide and methyl propyl trisulfide dipropyle trisulfide. Onion contains active compounds such as allyl propyl disulfide along with other active sulfur-containing compounds	[62–67]

*Allium sativum*	Bulb	5 ml/kg	X-ray 525 kv/s	Rats/biochemical parameters	Anti-oxidative and free radical scavenging	It is rich in c-glutamylcysteine and other sulfur-containing compounds giving a characteristic flavor. However, additional constituents of garlic include a wide range of primary and secondary nonsulfur biomolecules, such are steroidal glycosides, essential oil, flavonoids, anthocyanins, lectins, prostaglandins, fructan, pectin, adenosine, vitamins B1, B2, B6, C and E, biotin, nicotinic acid, fatty acids, glycolipids, phospholipids and essential amino acids	[67,71–81]

*Capsicum annuum*	Pericalps	0.1–10 μg/ml	2 Gy	Human lymphocytes/genotoxicity, superoxide radical scavenging	Superoxide radical scavenging activity	*Capsicum* contains many chemicals, including water, fixed (fatty) oils, steam-volatile oil, carotenoids, capsaicinoids, resin, protein, fibre and mineral elements. Red bell peppers contain 280 ug/gm total carotenoids. Capsanthin accounts for 60% of the total carotenoids. They also contain 11% β-carotene and 20% capsorubin. Capsanthin is acylated with C12–18 saturated fatty acids	[89–91]

*Centella asiatica*	Whole plant extract	100 mg/kg	8 Gy	Mice/survival, body weight, membrane damage	Antioxidant activity	Triterpenoids include asiatcoside, centelloside, madecossoside, thankuniside, isothankunic acid, centellose, asiatic, centellic and madecassic acids and brahmoside, brahminoside, brahmicacid, the structure of their genin, brahmic acid (2,6-hydroxy, 23-hydroxy-methyl ursolic acid) Asiaticoside and madecossoside. The fatty oil consists of glycerides of palmitic, stearic, lignoceric, oleic, linoleic and linolenic acids. An alkaloid, hydrocotylin has been isolated from the dried plants. Asiaticoside, madecossoside and centelloside have been isolated from the plant parts. Flavanoids, 3-glucosylquercetin, 3-glucosylkaemferol and 7-glucosylkaemferol have been isolated from the leaves	[93–99]

*Citrus aurantium*	Ripe fruit	250–1000 mg/kg	1.5 Gy	Mice/genotoxicity	Anti-oxidative and free radical scavenging	The main flavonoids occurred in cultivated citrus species are flavanone glycosides, hespiridin and naringin accounting 5% of dry weight of leaves and fruits and exhibit string antioxidant activity	[102–104]

*Coleus aromaticus*	Leaves	5 μg/ml	0.5, 1, 2, 4 Gy	Chinese hamster fibroblast cells (V79)	Antioxidant activity	The report on the chemical constituents of the leaves of *C. aromaticus* indicated the presence of carvacrol, thymol, eugenol, chavicol, ethyl salicylate, chlorophillin, flavonoids (cirsimaritin) and β-sitosterol-β-D-glucoside	[105–107]

*Coriandrum sativum*	Seeds	300 mg/kg 42 days	4 Gy	Rats/biochemical parameters	Free radical scavenging	Phytochemical constituents of *C. sativum* seeds showed the presence of polyphenols (rutin, caffeic acid derivatives, ferulic acid, galic acid and chlorogenic acid), flavonoids (quercetin and isoquercetin) and β-carotenoids. Seeds of coriander essential oils, however, leaves contain caffeic acid and flavonoids apart from volatile oils	[110–113]

*Crocus sativus*	Fridge dried extract	40 mg/kg	2 Gy	Mice/chromosomal damage; tissue biochemistry	Antioxidant activity	The major components of saffron are cis- and trans-crocins, which are glucosyl esters of 8,8′-diapocarotene-8,8′-dioic acid (crocetin), one of the few families of carotenoids that are freely soluble in water. It also contains safranal, which is a monoterpene aldehyde, and picrocrocin, which is a glycosidic precursor of safranal. Saffron is known to contain about 150 volatile and aromatic compounds including terpenes, terpene alcohol and their esters	[114–121]

*Curcuma longa*	Curcumin	5, 10 and 50 μg/ml	^131^I (100 μCi/1.5 ml)	Human lymphocytes/genotoxicity	Antioxidant activity	The chemical study of different samples of turmeric has yielded essential oil (4.2–14%), fatty oil (4.4–12.7%) and moisture (10–12.0%). It has been demonstrated that the presence of three major constituents curcumin (diferuloylmethane), p-hydroxycinnamoyl(feruloyl)methane and p, p’-dihydroxydicinnamoylmethanel. Oil has the components such as sesquiterpene, ketones and alcohols	[123–127]

	*C. longa* extract	200 mg/kg	6.5 Gy	Rat/biochemical parameters	Antioxidant activity		

*Cymbopogon citratus*	Whole plant extract	0.1%	2.5k Gy	Chicken meat	Antioxidant activity	The chemical composition of the essential oil of *C. citratus* consist of hydrocarbon terpenes, alcohols, ketones, esters and aldehydes. The essential oil mainly composed of citral, which is a mixture of two stereoisomeric monterpene aldehydes; the trans isomer geranial and cis isomer neral. It has been reported to contain flavonoids and phenolic compounds such as luteolin, quercetin, kampferol and apigenin glycosyl derivatives of the flavones apigenin and luteolin have been identified in infusions of the lemon grass leaves	[128–136]

*Elettaria cordamomum*	Ground dried fruit	2%	6 Gy	Albino rat/biochemical assay	Antioxidant activity	The volatile oil contains about 1.5% α-pinene, 0.2% β-pinene, 2.8% sabinene, 1.6% myrcene, 0.2% α-phellandrene, 11.6% limonene, 36.3% 1,8-cineole, 0.7% γ-terpinene, 0.5% terpinolene, 3% linalool, 2.5% linalyl acetate, 0.9% terpinen 4-ol, 2.6% α-terpineol, 31.3% α-terpinyl acetate, 0.3% citronellol, 0.5% nerd, 0.5% geraniol, 0.2% methyl eugenol and 2.7% trans-nerolidol. The cardamom aroma is produced by a combination of 1,8-cineole and α-terpinyl acetate	[139–141]

*Illicium verum*	Whole plant extract	0.1%	2.5 kGy	Chicken meat	Antioxidant activity	Anisyl acetone and benzenecarboxylic acid were identified as the main phenolic components present in aqueous fraction of *I. verum*	[136,142]

*Lavandula angustifolia*	Oil	20 μl; 40 μl	UV, 10, 20 30 Gy	Cell free assay/EPR, DPPH, reducing power assay	Antioxidant activity and free radical scavenging	A total of 47 compounds representing 98.4–99.7% of the oils were identified. 1,5-Dimethyl-1-vinyl-4-hexenylbutyrate was the main constituent of essential oil (43.73%), followed by 1,3,7-octatriene, 3,7-dimethyl- (25.10%), eucalyptol (7.32%) and camphor (3.79%)	[43,143–145]

*Mangifera indica*	Stem bark	5–100 μg/ml	5 Gy	Lymphoblastoid cells/DNA damage, protection and repair processes	Antioxidant activity	The bark is reported to contain protocatechic acid, catechin, mangiferin, alanine, glycine, γ-aminobutyric acid, kinic acid, shikimic acid and the tetracyclic triterpenoids cycloart-24-en-3β,26diol, 3-ketodammar-24 (E)-en-20S,26-diol, C-24 epimers of cycloart-25 en 3β,24, 27-triol and cycloartan-3β,24,27-triol	[147–149]

*Mentha piperita*	Oil	40 μl	8 Gy	Swiss albino mice/hematological and serum biochemistry	Antioxidant activity and free radical scavenging	Mentha extracts have antioxidant properties due to the presence of eugenol, caffeic acid, rosmarinic acid and α-tocopherol. Caffeic acid, rosmarinic acid, eriocitrin, luteolin-7-O-glucoside were identified as primary radical scavengers. It also contains phenolic acids, flavonoids and s-carvone	[15,151–158]

	Leaf extract	1000 mg/kg	8 Gy	Mice/survival assay, cytogenetic damage, testicular and intestinal damage	Antioxidant activity and free radical scavenging		

*Murraya Koenigii*	Leaf extract	100 mg/kg	4 Gy	Swiss albino mice/GSH, LPO	Antioxidant activity	Leaves are aromatic and contain proteins, carbohydrates, fiber, minerals, carotene, nicotinic acid and vitamin C. The leaves contain high amount of oxalic acid, leaves also contains crystalline glycosides, carbazole alkaloids, koenigin and resin. Fresh leaves contain volatile oil rich in vitamin A and calcium. It also contains girinimbin, iso-mahanimbin, koenine, koenigine, koenidine and koenimbine. Mahanimbicine, bicyclomahanimbicine, phebalosin, coumarine as Murrayone imperatoxin etc are isolated from leaves Triterpenoid alkaloids – cyclomahanimbine and tetrahydromahanmbine are present in the leaves. Alkaloids-murrayastine, murrayaline, pypayafolinecarbazole have been reported	[160–163,243]

*Myristica fragrance*	Seed	10 mg/kg	6, 8, 10 Gy	Swiss albino mice/survival assay, tissue biochemistry	Free radical scavenging	The chemical constituents include myristicin, lignan and eugenol. The essential oil of nutmeg contains mainly sabinene (15–50%), α-pinene (10–22%) and β-pinene (7–18%), with myrcene (0.7–3%), 1,8- cineole (1.5–3.5%), myristicin (0.5–13.5%), limonene (2.7–4.1%), safrole (0.1–3.2%) and terpinen4-ol (0–11%)	[164–167]

*Nigella sativa*	As gelatin capsule (450 mg)	250 mg/kg	8 Gy	Male albino rats/biochemical & hematological parameters	Oxidative stress	The most important active compounds of black seeds are thymoquinone, thymohydroquinone, dithymoquinone, p-cymene, carvacrol, 4-terpineol, t-anethol, sesquiterpene longifolene α-pinene and thymol among others. Seeds also contain alkaloids as isoquinoline and pyrazol ring bearing alkaloids. Additionally, *N. sativa* seeds contain α-hederin, a water soluble pentacyclic triterpene and saponin	[171–174]

*Ocimum sanctum*	Leaves	10 mg/kg	1–6 Gy	Mice/chromosomal damage	Free radical scavenging	Whole plant extract contains flavonoids, alkanoids, saponins, phenols, anthocynins, triterpenoids, tannins. Leaf extract contains flavonoids, alkanoids, saponins, tannins, phenols, anthocynins, terpenoids, steroils	[177–181]

			4.5 Gy	Mice/glutathione and antioxidant enzymes	Antioxidant activity		

*Origanum vulgare* L.	Dried powder of the plant	12.5, 25, 50 & 100 μg/ml	^131^I (20 μCi/ml)	Human lymphocytes/Micronuclei frequency	Free radical scavenging	Antioxidants present in oregano are rosmarinic acid, caffeic acid, flavomoids and derivatives of phenolic acids and α-tocopherol. Also rosmarinic acid methyl ester, oregano-A and oregano-B acts as antioxidants	[12,18,185,186]

	Leaf extract	100, 200 mg/kg	3 Gy	Mice/bone marrow cells	Anti-oxidative and free radical scavenging		

*Piper longum*	Dried powdered Fruits	400 mg/kg	6Gy	Mice/WBCs, bone marrow cells/GPT, ALP, LPO, GSH	Not known	Chemical studies have shown that the genus *Piper* has many components including unsaturated amides, flavonoids, lignans, aristolactams, long and short chain esters, terpenes, steroids, propenylphenols and alkaloids. The essential oils of ten Piperaceae species shown that the most frequently identified compounds were sesquiterpenes. However, the nonoxygenated monoterpenes (Z)p-ocimene, a-pinene and b-pinene were prevalent as well. A biosynthetic approach showed that the most common sesquiterpenes identified, E-caryophyllene and germacrene D, have the E, E-farnesyl-PP as fundamental precursor and only two were originated from E, Z-farnesyl-PP reactions (a-copaene and d-cadinene)	[188–193]

*Plumbago rosea*	Root extract	75 mg/kg	10 Gy	Experimental mouse tumor; S-180, Ehrlich ascites carcinoma	Radiosensitization for tumor killing effect	It has been reported that roots of *P. rosea* contains several naphthoquinonoids and their derivatives and flavonoids. The chemical constituents include plumbagin, palmitic acid and myricyl palmitate from petrol extract, and plumbagic acid lactone, ayanin and azaleatin from ethyl acetate extract of roots	[196–200]

*Rosmarinus officinalis* L.	Leaves	1000 mg/kg	6 Gy	Mice/liver and blood biochemistry	Free radical scavenging	It contains antioxidants such as carnosonic acid, carnosol, rosmarinic acid, rosmanol, isorosmanol and epirosmanol	[204,205]

*Salvia officinalis* L.	Leaves	2 gm/150 ml water	6 Gy	Rat/brain biochemistry	Antioxidant activity	Antioxidants present are salvinoloc acid (dimer of rosmarinic acid), carnosol, carnosic acid, rosmarinic acid, rosmanol, isorosmanol and epirosmanol	[6,8,209–211]

*Syzygium aromaticum*	Oil	200 mg/kg	7 Gy	Albino rat/liver and serum biochemistry	Anti-oxidative and free radical scavenging	Clove oil is an essential oil from the dried flower buds, leaves and stems of the tree *S. aromaticum* The main constituents of the essential oil are phenylpropanoids such as carvacol, thymol, eugenol and cinnaldehyde	[213,214]

*Syzygium cumini*	Leaf extract	0–100 μg/ml	3 Gy	Human lymphocytes/micronuclei induction	Free radical scavenging	The plant has been reported to poses acetyl oleanolic acid, triterpenoids, ellagic acid, isoquercitin, quercetin, kaempferol and myricetin in different concentrations	[94,216,217]

*Valeriana wallichii*	Root Extract	25, 50 or 100 μg/ml	2 Gy, 5 Gy and 150 Gy	Cultured human fibroblast cells/plasmid DNA	Free radical scavenging	Its rhizome and root contains volatile oil (valerianic oil) which is composed of alkaloids, boryl isovalerianate, chatinine, formate, glucoside, isovalerenic acid, 1- camphene, 1-pinene, resins, terpineol and valerianine. From the rhizomes, some important compounds, such as citric acid, malic acid, maliol, succinic acid and tartaric acid have been isolated	[220–222]

*Withania somnifera*	Root extract	100 mg/kg	6 Gy	Albino rats/hepatic biochemistry	Antioxidant activity	The extract of *W. somnifera* is a complex mixture of a large number of phytochemicals including phenolic compounds and flavonoids. However, the pharmacological effect of the roots of *W. somnifera* is attributed to withanolides. Withanolides are a series of naturally occurring steroids containing a lactone with a side chain of nine carbons, generally attached to C-17	[224–227]

*Zingiber officinale*	As tablet (400 mg)	250 mg/kg	8 Gy	Male albino rats/biochemical and hematological study	Oxidative stress	Gingerol-related compounds such as gingerol, shagaols, gengediols, zingerone, dehydrozingerone, gingerinone and diarylheptanoids accord antioxidant capacity to ginger rhizome. Geranial, camphene, p-cineole, α-terpineole, zingiberene and petandeconoic acid were major components of essential oil However, eugenol and zingerone were majoe components in ethanol oleoresin and methanol, CCl4 and isooctane oleoresin, respectively	[21,25,27,67,174,232–235]

	Fresh ginger extract	5 ml/kg	X-ray 525 kv/s	Rats/biochemical parameters	Anti-oxidative and free radical scavenging		

EPR: Electron paramagnetic resonance; GSH: Reduced glutathione; LPO: Lipid peroxidation; WBC: White blood cell.

The radioprotective effects of *A. conyzoides* extract were studied in mice. An optimum dose of 75 mg/kg was found to be effective against radiation doses ranging from 6 to 11 Gy and resulted in reduction in gastrointestinal- and bone marrow-associated death in mice. *In vitro* studies showed *A. conyzoides* extract was also effective in scavenging DPPH radicals suggesting free radical scavenging mechanism of radioprotection [56].

### *Allium cepa*

*Allium cepa*, belonging to the family Liliaceae that occurs worldwide, is a bulbous plant. India, China and the US are the leading producer [57,58]. Traditionally, it is used for the treatment of stomach ache, throat infection and hepatitis, and has properties such as antioxidant, antihyperglycemic, antihypertentive and anti-asthamatic [59–61]. Among the many phyto-active constituents documented, the essential oil of *A. cepa* contains compounds such as 3-1,8-cineole, L-linalool and camphordare and has been thoroughly investigated. Besides this, the onion bulb contains Kaempferol, β-sitosterol, ferulic acid, myritic acid and prostaglandins. Flavonoids and tannins are also present in *A. cepa*. Quercetin, quercetin 4-glucoside, taxifolin, taxifolin 7-glucoside and phenylalanine have been isolated from the bulb. The major sulfur compounds are dimethyl trisulfide, propenyl propyl disulfide, dipropyl disulfide, propenylmethyldisulfide and methyl propyl trisulfide dipropyle trisulfide. Onion contains active compounds such as allyl propyl disulfide along with other active sulfur-containing compounds [62–66]. Radiation protection and antioxidative effects of onion extract were studied in albino rats. Biochemical parameters were assessed such as alanine aminotransferase, superoxide dismutase and catalase in liver, kidney and heart. It was concluded that onion extract has significant radioprotective activity [67].

### *Allium sativum*

*Allium sativum* L. belongs to the genus *Allium*. The family Alliaceae has around 780 species based on new internal transcribed spacer (ITR) region of nuclear ribosomal DNA classification [68,69]. It is widely dispersed over the warm-temperate and temperate zones of the northern hemisphere. Today, it is grown in a number of countries and the leading producers are India, China and Korea [70]. It is rich in c-glutamylcysteine and other sulfur-containing compounds giving a characteristic flavor. However, additional constituents of garlic include a wide range of primary and secondary non-sulfur biomolecules, such as steroidal glycosides, essential oil, flavonoids, anthocyanins, lectins, prostaglandins, fructan, pectin, adenosine, vitamins B1, B2, B6, C and E, biotin, nicotinic acid, fatty acids, glycolipids, phospholipids and essential amino acids [71–81].

*A. sativum* extract has demonstrated radioprotective effects in mice and was found to be effective in significantly reducing the micronuclei frequencies induced by radiation [82]. A dose-dependent effect was evaluated on the frequencies of damaged cells and chromosomal aberrations, and it was recommended that administration of the extract for 30 days is vital for mitigating the clastogenic effects of genotoxicants [83]. Recent investigations showed that aged garlic is superior to fresh garlic as far as antiglycation and antioxidant activities are concerned [84].

### *Capsicum annuum*

*Capsicum annuum* L. belongs to the genus *Capsicum* of family Solanaceae [85], and is native to southern North America and northern South America [86]. Its fruit characteristics have been widely used for taxonomy [87] and have range greatly in type, colour, shape, taste and biochemical constituents [88]. Capsicum contains many chemicals, including water, fixed (fatty) oils, steam-volatile oil, carotenoids, capsaicinoids, resin, protein, fibre and mineral elements. Red peppers contain 280 μg/gm total carotenoids. Capsanthin accounts for 60% of the total carotenoids. They also contain 11% β-carotene and 20% capsorubin. Capsanthin is acylated with C12–18 saturated fatty acids [89,90].

The phenolic glycosides of *C. annum* L. were evaluated for their radioprotective effects, and oxidative damage induced by X-radiation was studied on human lymphocytes. Although these compounds showed less antiradical properties they had higher radioprotective ability, and no cytotoxicity was observed. Therefore, it was advocated that superoxide radical scavenging could be the favored method to screen compounds for their possible radioprotective ability [91].

### *Centella asiatica*

*Centella asiatica* belongs to family Umbellifere (Apiaceae). It is a herb found all over India and also in tropical and subtropical countries [92]. It contains triterpenoids such as asiatcoside, centelloside, madecossoside, thankuniside, isothankunic acid, centellose, asiatic, centellic and madecassic acids. The other constituents includes brahmoside, brahminoside and brahmic acid. The structure of genin and brahmic acid has been elucidated as 2,6-hydroxy, 23-hydroxy-methyl ursolic acid. The fatty oil consists of glycerides of palmitic, stearic, lignoceric, oleic, linoleic and linolenic acids. An alkaloid, hydrocotylin, has been isolated from the dried plants. Asiaticoside, madecossoside and centelloside have been isolated from the plant parts. Flavanoids, 3-glucosylquercetin, 3-glucosylkaemferol and 7-glucosylkaemferol have been isolated from the leaves [93–97].

*C. asiatica* extract at 100 mg/kg body weight was effective in mice against radiation (8 Gy)-induced loss in body weight, and in survival [98]. It was reported that *C. asiatica* offered protection against radiation to DNA as well as membranes. The proposed mechanism for this was by antioxidant function [99].

### *Citrus aurantium*

*Citrus aurantium* belongs to the family Rutaceae. The essential oil of *C. aurantium* L. var. amara possess antianxiety and motor relaxant effects in rats and mice [100,101]. The main flavonoids occurring in cultivated citrus species are flavanone glycosides, hespiridin and naringin, accounting for 5% of dry weight of leaves and fruits. These exhibit string antioxidant activity [102,103]. Citrus extract at different doses (250, 500 and 1000 mg/kg) have shown radioprotective effects against 1.5 Gy γ-irradiation in mouse bone marrow; however, 250 mg/kg dose was found to be the optimum dose, providing 2.2-fold protection. Radioprotective activity was assigned to the flavonoids contained in citrus extract [104].

### *Coleus aromaticus*

*Coleus aromaticus*, belonging to family Lamiaceae, is native to India and the Mediterranean and possesses various medicinal values. The report on the chemical constituents of the leaves of *C. aromaticus* indicated the presence of carvacrol, thymol, eugenol, chavicol, ethyl salicylate, chlorophillin, flavonoids (cirsimaritin) and β-sitosterol-β-D-glucoside [105,106]. Radioprotective potential was evaluated *in vitro* and *in vivo* for *C. aromaticus* extract. In cell-free assay, the extract has been shown to have radical scavenging activity and in V79 cells frequencies of micronuclei were evaluated against 0.5, 1, 2 and 4 Gy doses of γ-radiation. Both the assays demonstrated that the extract had antioxidant, anticlastogenic and radioprotective properties [107].

### *Coriandrum sativum*

*Coriandrum sativum* L. belongs to the family Umbelliferae (Apiaceae), commonly called as coriander. It is widely cultivated in the Middle East, Latin America, Africa and Asia [108]. Seeds as well as leaves have been used for flavoring food since ancient times [109]. Phytochemical constituents of *C. sativum* seeds showed the presence of polyphenols (rutin, caffeic acid derivatives, ferulic acid, galic acid and chlorogenic acid), flavonoids (quercetin and isoquercetin) and β-carotenoids. Seeds of coriander mainly contain essential oils; however, leaves contain caffeic acid and flavonoids apart from volatile oils [110,111].

The radio protective effect of coriander seeds against whole-body γ-irradiation was studied in rats. Treatment with coriander seed extract was effective in preventing radiation-induced biochemical changes in serum and significantly improved the antioxidant status in liver and kidney of rats. It is suggested that scavenging of free radicals was a possible mechanism of protection [112]. Ethanol extract of *C. sativum* was effective against Ultraviolet B (UVB)-induced skin photoaging *in vitro* and *in vivo*. Thinner epidermal layers and denser dermal collagen fibers was observed in treated mice, which also had lower MMP-1 levels and higher procollagen type I levels, thus suggesting the ability of *C. sativum* extract to prevent UVB-induced skin photoaging [113].

### *Crocus sativus*

*Crocus sativus* L. belongs to family Iridaceae and is known for its dried red stigma, popularly called as saffron and cultivated in Iran, India and Greece. The major components of saffron are cis- and trans-crocins, which are glucosyl esters of 8,8′-diapocarotene-8,8′-dioic acid (crocetin), one of the few families of carotenoids that are freely soluble in water. It also contains safranal, which is a monoterpene aldehyde, and picrocrocin, which is a glycosidic precursor of safranal. Saffron is known to contain around 150 volatile and aromatic compounds including terpenes, terpene alcohol and their esters. Safranal is considered to be responsible for its fragrance [114–120].

Pretreatment with freeze-dried saffron extract resulted in significant protective effects against radiation-induced genotoxic damage in mouse bone marrow; it reduced the level of lipid peroxidation, and resulted in an increase in glutathione content and activity of glutathione S-transferase, glutathione peroxidase and catalase liver and brain tissues of mice. However, in histopathological study of intestinal cells and male germ cells, there was nominal impact of saffron extract on radiation-induced damage [121].

### *Curcuma longa*

*Curcuma longa* L. belonging to family Zingiberaceae is a cultivated plant of the Asian tropical region. It is popularly called turmeric, and is used as a coloring and flavoring agent for food. It also has medicinal value, with aromatic, stimulant and carminative properties. Combination of slaked lime with turmeric acts as traditional cure against sprains and swellings due to injury [122]. The chemical study of different samples of turmeric has yielded essential oil (4.2–14%), fatty oil (4.4–12.7%) and moisture (10–12.0%). It has been demonstrated that there are three major constituents curcumin, p-hydroxycinnamoyl(feruloyl)methane and p,p’-dihydroxydicinnamoylmethanel. Its oil has components such as sesquiterpene, ketones and alcohols [123–125].

It has been suggested that there exists protective effects of curcumin against genetic damage as well as against the side-effects induced by ^131^I administration in terms of micronuclei frequency in human lymphocytes [126]. Radioprotective effects of *C. longa* extract were studied in rats against γ-irradiation, pre- and post-treatment the extract was found to be effective in modulating the levels of inflammatory cytokines, trace elements and the protein levels of SOD-1 and PRDX-1. Thus, modulation of antioxidant enzymes was held responsible for conferring radioprotection by *C. longa* extract [127].

### *Cymbopogon citratus*

*Cymbopogon citratus* (family Poaceae), commonly called lemon grass, is a widely used herb in tropical countries such as southeast Asia and Africa. The essential oil of the plant is used in aromatherapy and as a flavoring ingredient in herbal beverages. The chemical composition of the essential oil of *C. citratus* consists of compounds such as hydrocarbon terpenes, alcohols, ketones, esters and aldehydes. The essential oil is mainly composed of citral, which is a mixture of two stereoisomeric monterpene aldehydes; the trans-isomer geranial and cis-isomer neral. It has been reported to contain flavonoids and phenolic compounds such as luteolin, quercetin, kampferol and apigenin. Glycosyl derivatives of the flavones apigenin and luteolin have been identified in infusions of the lemon grass leaves [128–135].

Aqueous extract of *C. citratus* showed antioxidant and radioprotective properties. The extract was effective in reducing lipid peroxidation in irradiated minced chicken meat, able to scavenge DPPH and superoxide radicals at low concentrations, and protect DNA damage induced by radiation in pBR322 plasmid [136].

### *Elettaria cordamomum*

*Elettaria cordamomum* is commonly called cardamom and belongs to the family Zingiberaceae. It has light green pods and has aromatic dried fruits usually used in food preparation as well as for health benefits [137]. Cardamom oil has cosmetic values due to its cooling effects and is known to possess antioxidant activity [138]. The volatile oil contains about 1.5% α-pinene, 0.2% β-pinene, 2.8% sabinene, 1.6% myrcene, 0.2% α-phellandrene, 11.6% limonene, 36.3% 1,8-cineole, 0.7% γ-terpinene, 0.5% terpinolene, 3% linalool, 2.5% linalyl acetate, 0.9% terpinen 4-ol, 2.6% α-terpineol, 31.3% α-terpinyl acetate, 0.3% citronellol, 0.5% nerd, 0.5% geraniol, 0.2% methyl eugenol and 2.7% trans-nerolidol. The cardamom aroma is produced by 1,8-cineole and α-terpinyl acetate [139,140]. Cardamom has been evaluated for its radioprotective effects against γ-irradiation in rats, and found to afford protection against radiation-induced oxidative damage in liver and heart tissues [141].

### *Illicium verum*

*Illicium verum*, commonly known as Star anise, belongs to the family Illiciaceae and is native to China and Vietnam. The essential oil has confectionary application as a flavoring agent and industrial application in the preparation of Tamiflu to act against influenza virus. Anisyl acetone and benzenecarboxylic acid were identified as the main phenolic components present in aqueous fraction of *I. verum* [142]. *I. verum* extract showed radioprotective effects in irradiated minced chicken meat by reducing lipid peroxidation. It has been demonstrated that the *I. vernum* extract plays a significant role not only in enhancing the flavor of food along with antioxidants activity but also it find application as food supplement for preventing oxidative damage in the processed food [136].

### *Lavandula angustifolia*

*Lavandula angustifolia* belongs to the family Lamiaceae. The genus *Lavandula* comprises approximately 35 species that have significant application in aromatherapy. A total of 47 compounds representing 98.4–99.7% of the oils were identified. 1,5-dimethyl-1-vinyl-4-hexenylbutyrate was the main constituent of essential oil (43.73%), followed by 1,3,7-octatriene, 3,7-dimethyl- (25.10%), eucalyptol (7.32%) and camphor (3.79%) [43,143,144]. *Lavandula angustifolia* oil was assessed for its radioprotective activity against UV radiation and γ-irradiation. EPR spectroscopy and UV- and γ-irradiated oil samples have shown excellent DPPH radical scavenging activity. It was suggested that, after appropriate UV- or γ-irradiation treatment, lavender oil may have use as radioprotector and antioxidant for possible application in cosmetic and pharmaceutical industry [145].

### *Mangifera indica*

*Mangifera indica* belongs to the family Anacardiaceae and is commonly called mango or aam in Hindi. It is medicinally important in Ayurveda. It has strong antioxidant, anti lipid peroxidation, immunomodulation, cardiotonic, hypotensive, wound healing, antidegenerative and antidiabetic activities that are pharmacologically and medicinally important [146]. It contains chemical contents such mangiferin, a polyphenolic antioxidant and a glucosyl xanthone. The bark is reported to contain protocatechic acid, catechin, mangiferin, alanine, glycine, γ-aminobutyric acid, kinic acid, shikimic acid and the tetracyclic triterpenoids cycloart-24-en-3β,26diol, 3-ketodammar-24 (E)-en-20S,26-diol, C-24 epimers of cycloart-25 en 3β,24, 27-triol and cycloartan-3β,24,27-triol [147,148]. The extract *M. indica* was evaluated for radioprotection in human lymphocytes and lymphoblastoid cells. Interestingly, it was noticed that that higher doses induced DNA damage in human lymphocytes and lymphoblastoid cells, without affecting the DNA repair ability. However, protection was observed against radiation induced DNA damage at lower doses of *M. indica* extract [149].

### *Mentha piperita*

*Mentha piperita*, commonly called peppermint, belongs to the family Labiatae and is a perennial herb that grows up to height of 30–90 cm. It is an aromatic, stimulant and carminative and employed for treating nausea, flatulence and vomiting [150,244]. Mentha extracts have antioxidant properties due to the presence of eugenol, caffeic acid, rosmarinic acid and α-tocopherol. Caffeic acid, rosmarinic acid, eriocitrin, luteolin-7-O-glucoside were identified as primary radical scavengers. It also contains phenolic acids, flavonoids and s-carvone [15,151,152]. Mentha oil was found to afford radioprotection to hematological parameters and phosphatases level in mice [153,154]. Treatment of *M. piperita* extract prior exposure to γ radiation in mice has been shown to provide protection in bone marrow cells; it significantly reduced the number of aberrant cells and different chromosomal aberrations in irradiated mice [155]. Also, *M. piperita* extract pretreatment was efficient in providing protection against hematopoietic injury in bone marrow, intestine and testis in mice [156–158].

### *Murraya Koenigii*

*Murraya koenigii* L. belongs to the family Rutaceae and is commonly known as Meethi neem or curry-leaf in Hindi, and is a native of south Asia. It is found almost everywhere in the Indian subcontinent and has an aromatic nature, growing up to 6 m in height. It is especially cultivated for its aromatic leaves [159]. Leaves are aromatic and contain proteins, carbohydrates, fiber, minerals, carotene, nicotinic acid and vitamin C. The leaves contain high amount of oxalic acid and also contain crystalline glycosides, carbazole alkaloids, koenigin and resin. Fresh leaves contain yellow-colored volatile oil that is also rich in vitamin A and calcium. It also contains girinimbin, iso-mahanimbin, koenine, koenigine, koenidine and koenimbine. Mahanimbicine, bicyclomahanimbicine, phebalosin, coumarine as murrayone imperatoxin etc are isolated from leaves. Triterpenoid alkaloids-cyclomahanimbine and tetrahydromahanmbine are present in the leaves. Alkaloids-murrayastine, murrayaline, pypayafolinecarbazole have been reported in the leaves of *M. koenigii* [160–162,243]. The radioprotective effects of *M. koenigii* leaf extract was evaluated against 4 Gy γ-irradiation in liver of mice. The leaf extract itself was effective for significantly increasing reduced glutathione (GSH) content and antioxidant enzyme levels in liver as well as it reduced the radiation induced decrease in lipid peroxidation, thus indicating the antioxidant properties of extract possibly contributing for radioprotection [163].

### *Myristica fragrance*

*Myristica fragrance* belonging to family Myristicaceae is commonly called nutmeg and known for its antifungal, hepatoprotective and antioxidant properties. The chemical constituents include myristicin, lignan and eugenol. The essential oil of nutmeg contains mainly sabinene (15–50%), α-pinene (10–22%) and β-pinene (7–18%), with myrcene (0.7–3%), 1,8-cineole (1.5–3.5%), myristicin (0.5–13.5%), limonene (2.7–4.1%), safrole (0.1–3.2%) and terpinen4-ol (0–11%) [164–166]. Seed extract of *M. fragrance* was investigated for radioprotective effects in mice; it produced a dose reduction factor of 1.3. Pretreatment of *M. fragrance* seed extract was effective in increasing the GSH content in liver and reduction of testicular lipid peroxidation level in mice. It was demonstrated that *M. fragrance* seed extract offers a great degree of radioprotection in terms of radiation-induced biochemical alterations and enhanced survival rate, suggesting its possible utility as radioprotector [167].

### *Nigella sativa*

*Nigella sativa* belongs to the family Ranunculaceae and is commonly called black seed. It is a widely used medicinal plant throughout the world [168]. The seeds of *N. sativa* and their oil have been widely used for centuries in the treatment of various ailments considered to be an important drug in the Indian traditional system of medicine such as Unani and Ayurveda [169,170]. The most important active compounds of black seeds are thymoquinone, thymohydroquinone, dithymoquinone, p-cymene, carvacrol, 4-terpineol, t-anethol, sesquiterpene longifolene α-pinene and thymol among others. Seeds also contain alkaloids as isoquinoline and pyrazol ring bearing alkaloids. Additionally, *N. sativa* seeds contain α-hederin, a water soluble pentacyclic triterpene and saponin [171]. Radioprotection by *N. sativa* extract and oil was studied in mice and rats. The extract of *N. sativa* was evaluated in mice to assess protection against radiation-induced damage [172]. *Nigella sativa* extract treatment showed significant reduction in lipid peroxidation and intracellular reactive oxygen species in splenocytes and increased the survival rate of irradiated animals, suggesting a radioprotective potential of *N. sativa*. Oral administration of *N. sativa* oil before irradiation resulted in significant increase in blood malondialdehyde, nitrate and nitrite levels [173] and antioxidant enzymes [174].

### *Ocimum sanctum*

*Ocimum sanctum* L. belongs to family Labiateae, commonly known as holy basil, tulsi or tulasi. It is an aromatic, small annual herb, growing up to 18 inches and thought to have originated in north-central India. It now grows native throughout the eastern world tropics [175,176]. Whole-plant extract contains flavonoids, alkanoids, saponins, phenols, anthocynins, triterpenoids and tannins. Leaf extract contains flavonoids, alkanoids, saponins, tannins, phenols, anthocynins, terpenoids and steroils [177–179]. The radioprotective activity of extract of *O. sanctum* was evaluated in mice through chromosomal aberration analysis. The treatment of mice with extract of *O. sanctum* prior to irradiation resulted in faster recovery and reduced percentage of chromosomal aberrations in bone marrow cells. It was demonstrated that extract of *O. sanctum* offered *in vivo* protection against radiation-induced chromosomal damage and suggested that free radical scavenging could be the probable mechanism action [180–182].

### *Origanum vulgare* L.

*Origanum vulgare* belongs to the family Labiatae, and is generally found as a wild pant in Europe and Iran. It is used for treating rheumatism, muscle and joint pain, sore and swellings as an external applicant. Oregano oil is employed to counter toothache [183,184]. Antioxidants present in Oregano are rosmarinic acid, caffeic acid, flavomoids and derivatives of phenolic acids and α-tocopherol. Also, rosmarinic acid methyl ester, oregano-A and oregano-B act as antioxidants [12,18]. Radioprotection by Oregano extract was studied in terms of internal irradiation – as well as external irradiation-induced genotoxicity in human lymphocytes and mouse bone marrow. The oregano extract treatment resulted in significant reduction of micronuclei frequencies in human lymphocytes and mouse bone marrow. Radical scavenging activity of oregano extract was studied by DPPH assay, which shown that it was effective in scavenging of DPPH-free radical in dose-dependent manner. Therefore, free radical scavenging appears to be a likely mechanism for radioprotection [185,186].

### *Piper longum*

*Piper longum* belongs to family Piperaceae, and is commonly known as Pipali in India. It is traditionally used as a medicine in Asia and the Pacific Islands for treating diseases such as gonorrhea, menstrual pain, tuberculosis, arthritis and is also used for analgesic, diuretic and muscle relaxant purposes [187]. Chemical studies have shown that the genus *Piper* has many components including unsaturated amides, flavonoids, lignans, aristolactams, long and short chain esters, terpenes, steroids, propenylphenols and alkaloids. The essential oils of ten Piperaceae species have shown that the most frequently identified compounds were sesquiterpenes. However, the nonoxygenated monoterpenes (Z) p-ocimene, a-pinene and b-pinene were prevalent as well. A biosynthetic approach showed that the most common sesquiterpenes identified, E-caryophyllene and germacrene D, have the E,E-farnesyl-PP as fundamental precursor and only two were originated from E,Z-farnesyl-PP reactions (a-copaene and d-cadinene) [188–192]. The radioprotective effects of fruit extract of *P. longum* were studied in mice. Extract treatment prevented the radiation-induced depletion of white blood cells in mice. Extract treatment was also effective in declining the radiation-induced increased levels of glutathione pyruvate transaminase, alkaline phosphatase and lipid peroxidation thus offering protection to mice against radiation induced damage [193].

### *Plumbago rosea*

*Plumbago rosea* L. belongs to the family Plumbaginaceae, is popularly called rakta chitrak, and is found abundantly wildly growing in India. It is variously used to treat diseases such as inflammation, skin diseases, gastric troubles and abdominal pain [194,195]. It has active ingredients such as plumbagin, naphthaquinone, fatty alcohols, tannins and sitosterol glycosides. It has been reported that roots of *P. rosea* contains several naphthoquinonoids and their derivatives and flavonoids. The chemical constituents include plumbagin, palmitic acid and myricyl palmitate from petrol extract, and plumbagic acid lactone, ayanin and azaleatin from ethyl acetate extract of roots [196–198]. The extract of *P. rosea* was evaluated for antitumor activity. It has been reported that the *P. rosea* extract possesses radiosensitizing effects and combined with radiation increases the tumor killing effect [199,200].

### *Rosmarinus officinalis* L.

*Rosmarinus officinalis* L. belongs to family Labiateae. It is an aromatic and medicinal herb largely found along mediterranean sea coasts and Himalayan region of India [201]. *Rosmarinus* leaves were found to have significant antioxidant properties and is extensively used in food industry due to its non toxicity and safety [202,203]. It contains antioxidants such as carnosonic acid, carnosol, rosmarinic acid, rosmanol, isorosmanol and epirosmanol [204]. *R. officinalis* leaf extract was evaluated for its ability to protect the liver of mice against radiation-induced histopathological alterations. Extract treatment showed a significant decrease in lipid peroxidation and increase in GSH content in mice and there was significant decrease in binucleated hepatic cells as compared with untreated irradiated animals [205].

### *Salvia officinalis* L.

*Salvia officinalis* belongs to the family Lamiaceae and is cultivated in several countries. It has remedial and household importance [206]. The fragrance and aroma might be due volatile and essential oil that consists of mixture of volatile compounds such as terpenes, triterpenoids, ursolic acid and oleanolic acid [207,208]. Antioxidants present are salvinoloc acid (dimer of rosmarinic acid), carnosol, carnosic acid, rosmarinic acid, rosmanol, isorosmanol and epirosmanol [6,8,209]. The aqueous extract of *S. officinalis* showed significant radioprotection against irradiation in rats. Extract treatment resulted in decreased lipid peroxidation, protein carbonyl and NO in brain tissues and increased SOD and CAT enzymes activities and GSH contents [210]. *S. officinalis* extracts hold antimicrobial, anticancer, antioxidant, anti-inflammatory and radioprotective properties probably due to presence of active polyphenolic compounds that contains aromatic rings with hydroxyl groups [211].

### *Syzygium aromaticum*

*Syzygium aromaticum* (eastern hemisphere) or *Eugenia caryophyllata* and *Eugenia aromaticum* (western hemisphere) belongs to family Myrtaceae and is a popular source of clove oil; the essential oil extracted from dried flower buds, leaves and stem of the tree [212]. Clove oil is applied externally for relieving pain and promotes healing. It has wide application in pharmaceutical as well as the fragrance and flavoring industries. Clove oil is an essential oil from the dried flower buds, leaves and stems of the tree *S. aromaticum*. The main constituents of the essential oil are phenylpropanoids such as carvacol, thymol, eugenol and cinnaldehyde [213]. The radioprotective effect of clove oil has been studied in rats on certain biochemical parameters against ionizing radiation. It has been demonstrated that the radioprotective effects of clove oil may be assigned to its capacity to reduce lipid peroxidation, strong reducing power and superoxide radical scavenging activity owing to presence of the polyphenol as well as trace element contents [214].

### *Syzygium cumini*

*Syzigium cumini* belongs to the family Myrtaceae and is also called *Syzigium jambolanum* or *Eugenia cumini*. It is grown throughout Asia, Africa and America, and also naturalized to Florida and Hawaii [215]. The plant has been reported to possess acetyl oleanolic acid, triterpenoids, ellagic acid, isoquercitin, quercetin, kaempferol and myricetin in different concentrations [94]. The radioprotective effects were studied in cultured human lymphocytes; it was noted that treatment with *S. cumini* leaf extract prior to radiation exposure resulted in significant decrease in micronuclei induction [216]. Radioprotective effects of *S. cumini* seed and leaf extracts were also studied in mice and found that pretreatment protected mice against radiation-induced sickness and mortality. The histopathological investigations showed that *S. cumini* leaf extract treatment prior to radiation exposure increased villus height, number of crypts and reduced goblet and dead cells [217].

### *Valeriana wallichii*

*Valeriana wallichii*, belonging to the family Valerianaceae and popularly known as Indian Valerian, occurs throughout the World. It mostly grows in mountain terrain of Himalaya and is used as an ingredient in various herbal medicines in Indian systems of medicine [218,219]. Its rhizome and root contains volatile oil (valerianic oil), which is composed of alkaloids, boryl isovalerianate, chatinine, formate, glucoside, isovalerenic acid, 1-camphene, 1-pinene, resins, terpineol and valerianine. From the rhizomes, some important compounds, such as citric acid, malic acid, maliol, succinic acid and tartaric acid have been isolated [220,221]. The root extract of *V. wallichii* was found to significantly protect against radiation-induced free radicals at 4 h after 5 Gy irradiation, reduced prolonged oxidative stress led increase in mitochondrial mass, enhanced reproductive viability of cultured cells and protected against radiation-induced DNA damage [222].

### *Withania somnifera*

*Withania somnifera*, belonging to the family Solanaceae and popularly known as Indian ginseng or ashwagandha, is a perennial plant with medicinal importance in Ayurveda [223]. The extract of *W. somnifera* is a complex mixture of a number of phytochemicals including phenolic compounds and flavonoids. However, the pharmacological effect of the roots of *W. somnifera* is attributed to withanolides. Withanolides are a series of naturally occurring steroids containing a lactone with a side chain of nine carbons, generally attached to C-17 [224–226]. The protective effects of root extract of *W. somnifera* against radiation-induced oxidative stress and DNA damage in liver were investigated in rats. *Withania somnifera* treatment prior to radiation exposure showed significant decrease in hepatic enzymes, hepatic nitrate/nitrite ratio, MDA levels and DNA damage. Also, significant increase in heme oxygenase activity, superoxide dismutase, glutathione peroxidase activities and glutathione content suggests a possible role of *W. somnifera* as a radioprotective agent through antioxidant function and heme oxygenase induction [227].

### *Zingiber officinale*

*Zingiber officinale* is commonly called ginger and belongs to the family Zingiberaceae. It is found in Asia and America. It is medicinal and aromatic and generally employed for culinary practices and remedial uses. The essential oil possesses antibacterial, antifungal and antiviral activities [228,229]. Antioxidant, anti-inflammatory and antinoceceptive properties have also been reported [230,231]. Gingerol-related compounds such as gingerol, shagaols, gengediols, zingerone, dehydrozingerone, gingerinone and diarylheptanoids accord antioxidant capacity to the ginger rhizome. Geranial, camphene, p-cineole, α-terpineole, zingiberene and petandeconoic acid were the major components of the essential oil. However, eugenol was major components in ethanol oleoresin, and in methanol, CCl4 and isooctane oleoresin the zingerone was major component [21,25,27,232–234]. Radioprotective effects of ginger extract were demonstrated in rats exposed to X-irradiation on the liver, kidney and heart. Results indicated that ginger extract had significant antiradiation activity [67]. Antioxidant status and antioxidant enzymes were studied in rats with pretreatment of ginger extract and whole-body irradiated with γ-radiation. Hematological parameters were found to have significant recovery with respect to radiation-induced damage [174]. Essential oil of ginger was also evaluated for its radioprotective effects in mice, yielding a dose reduction factor of 1.4. Ginger oil was found to be effective in restoring antioxidant status and reducing the cytogenetic damage in terms of chromosomal aberrations, micronuclei frequency and DNA damage in mice [235].

## Possible mechanisms of radioprotection by aromatic plants

It is a well-known fact that radiation is a powerful cytotoxic agent. Reactive oxygen species such as superoxide anion, singlet oxygen, hydroxyl radical, nitric oxide, hydrogen peroxide and peroxyl radicals are generated by ionizing radiation in biologic system through radiolysis of water and are accountable for the cellular injury caused to DNA and proteins [31–33]. The detrimental effects of radiation-induced alterations in biologic systems via reactive oxygen species generation play a crucial role as far as maintenance of metabolic homeostasis in the body and therefore, any disparity in homeostasis results in oxidative stress [38], which could be trounced by additional provision of naturally occurring, plant based antioxidants [39–41]. The extracts of aromatic plants contain several chemical constituents such as essential oils, plant phenolics such as phenolic acids, flavonoids, terpenes, tannins, stilbenes, lignans and vitamins. Among these, the essential oils contain constituents such as monoterpenes and diterpenes that possess antioxidant properties. The activity of cyclic monoterpene hydrocarbons with two double bonds has been found to be comparable to the activity of phenols; however, their antioxidant activities differ due to their composition and oxidation of the components. The correlation between antioxidant capacity and phenolics concentration was pragmatic in several research studies on determining antioxidant capacities of plants [236]. Phenolic compounds are the secondary metabolites of plants and involved in defense against ultraviolet radiation or aggression by pathogens, parasites and predators [237]. The chemical structure of phenolic compounds also affects their antioxidant effects. Phenolics have usually one or more aromatic rings that act as an extended conjugated aromatic system to delocalize an unpaired electron and one or more hydroxyl groups which donate a hydrogen atom or an electron to a free radical. Therefore, these phytoconstituents have most suitable structure for free radical scavenging activities. The polyphenols also act as reducing agents, hydrogen-donating antioxidants, singlet oxygen quenchers and metal chelators thus imparting their inherent antioxidant activity. On the other hand, flavonoids interfere with the propagation reactions of the free radical and formation of the radicals by chelating the transition metal. The α-Tocopherol, one of the best known vitamins confer protection to intracellular membranes mainly by quenching singlet oxygen and reacting with lipid peroxy radicals which consequently leads to abrogation of lipid peroxidation levels [238]. Ascorbic acid (Vitamin C) was found to work synergistically with tocopherols and other phenolic antioxidants. Carotenoids, such as β-carotene, lutein and zeaxanthin, are present in certain aromatic plants such as mints, oregano, balm, basil, sage and rosemary which have demonstrated antioxidant activity both in *in vitro* and animal experimental studies. Thus, antioxidant function and radical scavenging mechanism are the most likely mechanism of radioprotection by aromatic plants as suggested by several workers (Table 1).

## Merits, demerits & limitations

Due to their abundance, low cost and safety, aromatic plants hold significant promise and provide unique advantage for use as radioprotectors. These plants have varied antioxidant capacities which directly correlates with their chemical constituents thus have variable radioprotective properties [239]. It has been reported that carotenoids can protect against radiation but a high dose of single carotenoid entity induces lethal effects [240]. Therefore, quality control studies must be focused on proper elucidation of definite effects of the drug used with factors such as age, sex and species accounted for while performing preclinical assessment studies [241]. The use of plant-derived medication has limitations because only limited systematic studies are available for each plant product. Therefore, research must be directed toward acquiring knowledge about the safe use of plant-based drugs prior to their possible use [122]. However, studies on pharmacokinetics and pharmacodynamic properties including toxicity must be carried out [242]. Most of the studies reported are conducted on animal models or cell cultures and therefore, it is not easy to extend their validity in clinical settings – this poses a major hindrance [39]. It has been observed that crude extract is better than the isolated fractions; this may be due to the combined effects of so many different constituents that are responsible for its activity. The compound/extract must work in practice and not just be limited to laboratory. Plant material utilized as a specific compound or group of compounds should be well standardized, characterized and processed.

## Future perspective

The enhanced adverse effects of synthetic drugs and antibiotics has paved way toward utilization of natural products of plant origin in recent years in developed as well as developing countries. The present review explored the protection against radiation at cellular damage of approximately thirty aromatic plant extracts or plant-derived compounds. Most of the aromatic plant extracts or plant products have shown significant radioprotection in different model systems such as *in vivo*, *ex vivo* and/or *in vitro* for assessment of radiation-induced damage. The radical scavenging and antioxidant properties such as reduction in radiation-induced lipid peroxidation are some of the notable characteristics of aromatic plant extracts studied in various models of radiation insult. In most of the studies, it has been shown that while protecting against the detrimental effects of radiation, it also had capacity to significant increase survival rates in small animals exposed to radiation. Results of such studies point at possible application during radiotherapy as well as the possibility of finding application in treatment for victims of nuclear plant accidents or leakage, or radiation terrorism. Use of aromatic plants and their products has gained momentum globally during recent times with wide applications in the herbal drug industry. Natural resources such as wastelands and forests could serve as reservoir for the same. However, with the increasing burden on natural resources, alternatively, introduction of crops through cropping systems could help out to great extent with a check on activity including chemical composition. Also, the scientific community must be encouraged to focus studies to screen more and more aromatic compounds of plant origin for their different bioactivities, including radioprotection, and to explore the molecular mechanisms involved for the same.

**Executive summary**

- The enhanced adverse effect of synthetic drugs and antibiotics has paved way toward the utilization of natural products of plant origin unambiguously in recent years.
- The present review explored the protection against cellular damage resulting from radiation of approximately thirty aromatic plant extracts or plant-derived compounds.
- Many aromatic plant extracts or plant products have shown significant radioprotection in different model systems such as *in vivo*, *ex vivo* and/or *in vitro*.
- It is a well-known fact that radiation is a powerful cytotoxic agent. Reactive oxygen species such as superoxide anion, singlet oxygen, hydroxyl radical, nitric oxide, hydrogen peroxide and peroxyl radicals generated by ionizing radiation in biologic system causes cellular injury due to lesions in DNA and proteins.
- The radical scavenging and antioxidant properties such as reduction in radiation-induced lipid peroxidation are some of the notable characteristics of aromatic plant extracts studied in various models of radiation damage.
- In most of the studies, it has been shown that while protecting against the detrimental effects of radiation, there is a capacity to significantly increase the survival rates of small animals exposed to radiation. Results of such studies point out possible applications during radiotherapy and could find application in treatment for victims of nuclear plant accidents or leakage, or radiation terrorism.
- Aromatic plants and their products have gained momentum globally during recent times, with wide applications in the herbal drug industry.
- Natural resources such as wastelands and forests could serve as reservoir for the same. With the increasing burden on natural resources, alternatively, introduction of crops through cropping systems could help out to a great extent with checks on activity including chemical composition.
- Plant material utilized as a specific compound or group of compounds should be well standardized, characterized and processed.
- Also, the scientific community must be encouraged to focus studies on screening more and more aromatic compounds of plant origin for their different bioactivities, including radioprotection, and to explore the molecular mechanisms involved in the same.
